# Chromosome-Level Genome Sequence of the Black Koji Fungus Aspergillus luchuensis RIB2601

**DOI:** 10.1128/MRA.00384-21

**Published:** 2021-07-22

**Authors:** Kazuki Mori, Chihiro Kadooka, Atsushi Nishitani, Kayu Okutsu, Yumiko Yoshizaki, Kazunori Takamine, Kosuke Tashiro, Masatoshi Goto, Hisanori Tamaki, Taiki Futagami

**Affiliations:** a Cell Innovator Co., Ltd., Fukuoka, Japan; b Education and Research Centre for Fermentation Studies, Faculty of Agriculture, Kagoshima University, Kagoshima, Japan; c United Graduate School of Agricultural Sciences, Kagoshima University, Kagoshima, Japan; d Research Support Center, Institute for Research Promotion, Kagoshima University, Kagoshima, Japan; e Laboratory of Molecular Gene Technology, Faculty of Agriculture, Kyushu University, Nishi-ku, Fukuoka, Japan; f Department of Applied Biochemistry and Food Science, Faculty of Agriculture, Saga University, Saga, Japan; University of California, Riverside

## Abstract

Aspergillus luchuensis is used for the production of awamori and shochu, which are traditional Japanese distilled alcoholic beverages. Here, we determined the chromosome-level genome sequence of *A. luchuensis* RIB2601.

## ANNOUNCEMENT

The black koji fungus Aspergillus luchuensis is used for the production of awamori and shochu, which are traditional distilled alcoholic beverages indigenous to Japan (1–3). *A. luchuensis* was originally used to produce awamori in Okinawa, Japan’s southernmost prefecture; then, it was used to produce shochu (4). The black koji fungus plays an important role in supplying glycoside hydrolases for decomposing starch contained in the ingredients of awamori and shochu during the fermentation process (1–4). In addition, it excretes a large amount of citric acid, which can prevent the growth of contaminating microbes during fermentation (1–4).

Currently, the genome sequence of *A. luchuensis* NBRC 4314 (RIB2604) is available (5); however, there are a variety of strains of the black koji fungus (1–3). We previously studied an amylolytic enzyme-overproducing mutant of *A. luchuensis* RIB2601 (6); therefore, we sequenced the genome of RIB2601. Strain RIB2601 was cultivated in yeast extract-peptone-dextrose medium (2% [wt/vol] glucose, 1% [wt/vol] yeast extract, and 2% [wt/vol] peptone) overnight. Then, the retrieved mycelia were subjected to DNA extraction using DNAs-ici!-F (Rizo, Inc., Tsukuba, Japan). The genomic DNA of RIB2601 was sequenced with coverages of 37- and 336-fold using Oxford Nanopore Technologies (ONT) MinION and Illumina HiSeq 2000 instruments, respectively. ONT and Illumina sequencing libraries were prepared using the ligation 1D (SQK-LSK109) and Illumina TruSeq DNA sample prep kits, respectively. The ONT reads and Illumina reads were used for *de novo* assembly and error correction, respectively. The ONT reads were assembled using Canu v2.0 (7); then, the initial assembly and trimmed corrected ONT reads were reassembled using Flye v2.8-b1674 (8). The final assembly was polished using medaka v1.0.3 (9) with ONT reads, Pilon v1.23 (10) with ONT reads, and Pilon v1.23 (10) with Illumina reads. The genome sequence of RIB2601 was assembled into nine contigs, which consist of eight chromosomes and one mitochondrial DNA. In addition, we found telomeres on both ends of each chromosome sequence, thus indicating that we successfully sequenced the nearly complete genome sequence of RIB2601. Genome annotation of the obtained chromosomal contigs and mitochondrial contig was performed using the Funannotate v1.8.1 pipeline (11) and MFannot v1.1 (12), respectively. Gene prediction was performed using SNAP v2006-07-28 (13), AUGUSTUS v3.3.3 (14), GlimmerHMM v3.0.4 (15), and GeneMark-ES v4.61_lic (16) via the Funannotate v1.8.1 pipeline (11). For the analysis, transcriptome sequencing (RNA-seq) reads of strain RIB2601 (6) (Sequence Read Archive [SRA] accession numbers SRX2414184 to SRX2414186) were *de novo* assembled using Trinity v2.8.5 (17) and used for gene prediction with the sequence alignment tool HISAT v2.2.0 (18). The proteins were annotated using MEROPS v12.0 (19), UniProt v2020_05 (20), MIBiG v1.4 (21), Pfam v33.1 (22), and dbCAN2 v9.0 (23) (based on CAZy database v7/30/2020 [24]) with sequence alignment tools such as DIAMOND v2.0.6 (25) and HMMER v3.3.2 (26). The annotation was also performed using InterProScan v5.47-82.0 (27), eggNOG-mapper v1.0.3 (28) (for the EggNOG v4.5 database [29]), antiSMASH v5.1.2 (30), SignalP v4.1 (31), Phobius v1.01 (32), tRNAscan-SE v2.0.7 (33), and Barrnap v0.9 (34). The RIB2601 genome includes 35,508,746 bp with a GC content of 48.8% and is comprised of 11,553 predicted coding sequences and 287 tRNAs. The genome completeness was assessed using BUSCO v5.1.2 with the ascomycota_odb10 data set (35), resulting in 98.9% complete and single-copy, 0.2% complete and duplicate-copy, 0.3% fragmented-copy, and 0.6% missing benchmarking universal single-copy orthologs (BUSCOs). The details for each replicon are summarized in Table 1. The chromosome-level genome sequence will aid in subsequent genomics research of black koji fungi.

**TABLE 1:** Replicons of Aspergillus luchuensis strain RIB2601

Location^*a*^	GenBank accession no.	Size (Mb)	% GC	No. of CDS^*b*^	No. of rRNAs^*c*^	No. of tRNAs
Chr 1	AP024434.1	6.19	49.5	2,011	NA	53
Chr 2	AP024435.1	4.96	48.9	1,553	NA	46
Chr 3	AP024436.1	4.48	49.4	1,477	NA	33
Chr 4	AP024437.1	3.78	49.2	1,294	9 (70)^*d*^	18
Chr 5	AP024438.1	4.82	47.9	1,490	NA	30
Chr 6	AP024439.1	3.97	48.7	1,323	NA	40
Chr 7	AP024440.1	3.19	48.2	1,026	NA	14
Chr 8	AP024441.1	4.07	48.6	1,364	NA	39
MT	AP024442.1	0.03	26.4	15	1	26

a Chr, chromosome; MT, mitochondria.

b CDS, coding DNA sequences.

c NA, not applicable.

d The number of rRNA genes is not clear due to their highly repetitive structure. The number in parentheses indicates the estimated copy number based on the median per-base coverage.

### Data availability.

The nucleotide sequences of the *A. luchuensis* RIB2601 chromosomes and mitochondria have been deposited at DDBJ/ENA/GenBank under accession numbers AP024434 to AP024442. The raw sequence reads were deposited in the SRA under accession numbers DRX251232 and DRX251231.
